# Two Common Bean Genotypes with Contrasting Response to Phosphorus Deficiency Show Variations in the microRNA 399-Mediated *PvPHO2* Regulation within the PvPHR1 Signaling Pathway

**DOI:** 10.3390/ijms14048328

**Published:** 2013-04-16

**Authors:** Mario Ramírez, Gerardo Flores-Pacheco, José Luis Reyes, Ana Luz Álvarez, Jean Jacques Drevon, Lourdes Girard, Georgina Hernández

**Affiliations:** 1 Centro de Ciencias Genómicas, Universidad Nacional Autónoma de México (UNAM), Av. Universidad 1001, Cuernavaca 62210, Morelos, Mexico; E-Mails: gerryflores7@hotmail.com (G.F.-P.); analuz82@hotmail.com (A.L.A.); girard@ccg.unam.mx (L.G.); gina@ccg.unam.mx (G.H.); 2 Departamento de Biología Molecular de Plantas, Instituto de Biotecnología, UNAM, Av. Universidad 2001, Cuernavaca 62210, Morelos, Mexico; E-Mail: jlreyes@ibt.unam.mx; 3 Institut National de la Recherche Agronomique, UMR Eco&Sols-Ecologie Fonctionnelle & Biogéochimie des Sols & Agroécosystèmes, 2 Place Viala, Montpellier F34060, France; E-Mail: drevonjj@supagro.inra.fr

**Keywords:** *Phaseolus vulgaris*, common bean, phosphorus deficiency, post-transcriptional regulation by microRNAs, PvPHR1/PvmiR399 signaling pathway

## Abstract

Crop production of the important legume, the common bean (*Phaseolus vulgaris*), is often limited by low phosphorus (P) in the soil. The genotypes, BAT477 and DOR364, of the common bean have contrasting responses to P starvation. Plants from the BAT477 P deficiency tolerant genotype showed higher phosphate content and root biomass as compared to the DOR364 plants under P starvation. The PvPHR1 transcription factor-signaling pathway plays an essential role in the response to P starvation. *PvPHO2*, a negative regulator of this pathway, encodes an ubiquitin E2 conjugase that promotes degradation of P-responsive proteins and is the target gene of PvmiR399. *PvPHO2* is downregulated in BAT477 plants under P deficiency, while such a response is not observed in P-starved DOR364 plants. Five putative PvmiR399 binding sites were identified in the 5′ UTR region in both genotypes. While four sites showed an identical DNA sequence, the fifth (binding site of *PvPHO2* one) showed three base changes and higher complementarity scores in DOR364 as compared to BAT477. Modified 5′RACE experiments indicated that PvmiR399 binding and/or processing was affected in DOR364 P-starved plants. We propose that a less efficient cleavage of the *PvPHO2* mRNA directed by PvmiR399 would result in a higher PvPHO2-mediated degradation of P-responsive proteins in the DOR364 genotype with decreased P deficiency tolerance.

## 1. Introduction

Phosphorus (P) is an essential macronutrient for plant growth and development. It is a major component of fundamental macromolecules and is important for energy transfer and for the regulation of enzyme activity [1–3]. Plants acquire P as phosphates from the soil, but phosphate availability is limiting for plant growth and crop yield in more than 30% of the world’s arable land [4]. The application of P fertilizer can compensate for low P availability in cropping systems, but high phosphate input can cause severe environmental problems. Each year, millions of tons of phosphate are being mined from finite ground deposits that are gradually depleted. Taking into account the predicted growing demand for fertilizers to satisfy an increasing population, it has been calculated that P world reserves will last for around 50–100 years or even only until 2050 [5].

Plants have evolved diverse morphological, physiological and biochemical strategies to obtain adequate P amounts under limiting conditions [4]. Several P-deficiency responses are regulated at the transcriptional level [6–8]. Global transcriptome analyses have allowed for the identification of numerous responsive genes involved in adaptation to low P of different plant species, such as *Arabidopsis thaliana* (*Arabidopsis*) *Oryza sativa* (rice), *Lupinus albus* (white lupin) and *Phaseolus vulgaris* (common bean) [9–15]. In addition, genetic and molecular approaches have demonstrated P starvation signaling pathways controlled by different transcription factors (TF). In *Arabidopsis*, at least five TF—PHR1, WRKY75, ZAT6, BHLH32 and MYB62—with essential roles in low P signaling pathways have been characterized [16–19].

The *Arabidopsis PHR1* gene (*PHOSPHATE STARVATION RESPONSE 1*) [20], homologous to *PSR1* (*PHOSPHORUS STARVATION RESPONSE 1*) in *Chlamydomonas reinhardtii*[21], encoding a MYB protein is the best-characterized TF implicated in vascular plant P deficiency signaling. PHR1 is expressed in P sufficient conditions, and it is only slightly induced after sensing P deficiency. PHR1, localized to the nucleus, recognizes the imperfect palindrome, GNATATNC (P1BS or PHR1-binding sequence), that is present in the promoter regions of PHR1-controlled P-responsive genes, including genes involved in P remobilization, such as acid phosphatases and RNases, in P transport, such as *PTH1* and *PHO1*, in P homeostasis and in anthocyanin biosynthesis [20,22–24].

Three regulatory genes from the PHR1 signaling pathway, relevant for the regulation of P homeostasis, encoding for microRNA 399 (miR399), At4 and PHO2/UBC24 have been characterized. PHR1 positively regulates a miR399 precursor that is processed by Dicer-like 1 (DCL1) and Argonaute 1 (AGO1) proteins [25], giving rise to mature miR399 that is highly increased in P-depleted vascular tissues, shoots and roots [26]. Also, miR399 has been detected in the phloem sap of rapeseed (*Brassica napus*) and pumpkin (*Cucurbita maxima*) [27,28] and, thus, may serve as a systemic signal for P starvation [26]. During P starvation, miR399 recognizes and degrades its target mRNA, *PHO2/UBC4*, an ubiquitin E2 conjugase, that is a negative regulator of P-responsive genes, such as the high affinity P transporter, PHT1 [29–32]. PHR1 is also a positive regulator of the *At4* gene, from the *IPS1* gene family, that negatively regulates miR399 activity through a target mimicry mechanism [33]. A region within the *At4* sequence is complementary to miR399, but the pairing is interrupted by a mismatch loop at the miRNA cleavage site; thus, *At4* is not cleaved, but instead sequesters miR399, preventing the degradation of its target *PHO2*. This elegant mechanism enables the transient downregulation of *PHO2* by miR399 during P starvation, allowing not only a rapid response to starvation, but also a quick return to normal levels afterwards to prevent P toxicity [33]. Homologous genes of miR399, *PHO2* and *At4/IPS1* have been identified in other plant species, including rice, *Medicago truncatula* and the common bean [34–37]. The regulation of miR399 and *PHO2* is conserved among plants, thus highlighting the evolutional importance of the PHR1 signaling pathway in response to P deficiency.

The common bean (*Phaseolus vulgaris*) is the world’s most important grain legume for direct human consumption. It is widely grown in poor soils of Latin America and Africa, where P deficiency is perhaps the most limiting factor for symbiotic nitrogen fixation, resulting from the association with rhizobial bacteria, and for crop productivity [38]. Research from our group has identified a plethora of genes and metabolites that respond to P deficiency in common bean roots and root nodules [14,15]. In addition, a reverse genetic approach has led to demonstration of the essential role of the PvPHR1 signaling pathway in P-deprived common bean roots [38].

The biodiversity of *P. vulgaris* species is well documented, and genotypes that show differences in the ability to efficiently acquire and utilize P under P-limiting conditions have been characterized [38–42]. The latter includes the BAT477 and DOR364 genotypes that show contrasting responses to P deficiency. Both genotypes were developed at the International Center for Tropical Agriculture (CIAT) in Cali, Colombia. BAT477 is a drought tolerant breeding line that when grown in P-deficient field conditions, shows a good symbiotic N fixation capacity. Instead, DOR364, a disease-resistant genotype, when grown under P deficiency, forms smaller nodules and shows a much-reduced symbiotic N fixation capacity, due to low P use efficiency [40]. The latter is reflected in lower seed production of DOR364 plants compared with BAT477 plants grown under similar P starvation conditions [41,42]. Different reports suggest that the difference in P acquisition efficiency between these genotypes may be related to their ability to alter rhizosphere conditions that are known to influence the bioavailability of soil P, via the release of protons organic acids or phosphatase-like enzymes [7,43,44].

In this work, we aimed to explore possible factors that may influence or determine the contrasting P deficiency response in the BAT477 *vs.* DOR364 *P. vulgaris* genotypes. Our comparative analysis focused in the PvPHR1/PvmiR399 signaling pathway that, as we demonstrated, plays an essential role in P deficiency signaling in common bean roots. Our rationale was that a variation in the regulation/function of one or more of the key components from this pathway may be related to the differences in tolerance to P starvation between the two genotypes. Therefore, we did a comparative analysis of the expression levels of key regulatory genes of the PvPHR1/PvmiR399 signaling pathway. We then performed an *in silico* analysis of the *PvPHO2* gene DNA sequence from both genotypes to identify potential PvmiR399 binding sites, as well as their degree of complementarity. Furthermore, we experimentally validated the miR399-dependent cleavage position of *PvPHO2* from two of the five identified putative PvmiR399 binding sites: binding site 3, which is identical, and binding site 1, which shows differences in the degree of PvmiR399/*PvPHO2* complementarity between the two genotypes. The different regulation of *PvPHO2,* a negative regulator of the PvPHR1 pathway, between the BAT477 and DOR364 plants presented here, provides insights related to the contrasting P response of these *P. vulgaris* genotypes.

## 2. Results and Discussion

### 2.1. Comparative Phenotypic Analysis of BAT477 vs. DOR364 Plants under P Deficiency

**Figure 1:**
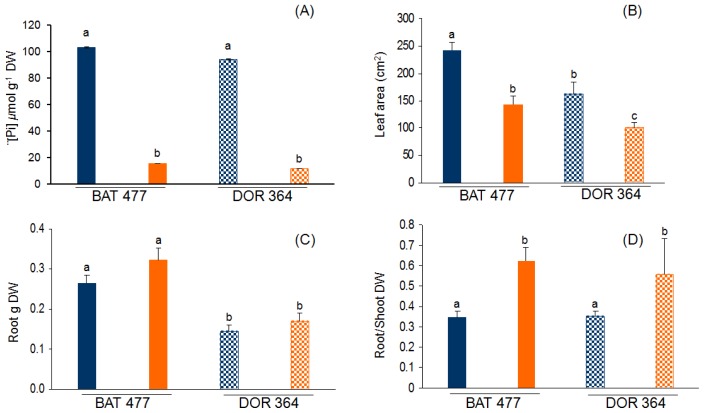
Effect of phosphorus (P) deficiency on common bean BAT 477 and DOR 364 genotypes. (**A**) soluble P_i_ content in leaves; (**B**) leaf area from fully expanded leaves; (**C**) root dry weight; (**D**) root to shoot dry weight ratio. Plants were grown for three weeks under P sufficient (blue bars) or in P deficient (orange bars) conditions. Values are the mean (±SE) from two independent experiments with eight replicates per experiment. Within each panel, bars marked with different letters represent significantly different means according to the statistical analysis (*p* ≤ 0.05).

The P deficiency (−P) treatment used consisted of 200-fold lower P_i_ content in the nutrient solution used to water common bean plants, as compared to the full-nutrient (control) condition. After three weeks of treatment, P starvation was evident in both BAT477 and DOR364 bean genotypes. The leaf P_i_ content was 6.8-fold lower in BAT477 plants and 8.5-fold lower in DOR364 plants grown in −P as compared to control condition (Figure 1A). While P_i_ content in leaves was similar in control plants from the two genotypes, 27.5% higher amount of P_i_ was observed in bean BAT477 plants as compared to bean DOR364 plants grown in −P conditions (Figure 1A). Common and well-characterized responses to P starvation of different plant species include decreased leaf area and increased root growth that results in a higher root to shoot weight ratio [7,45–47]. We performed a comparative analysis of such responses in the two common bean genotypes. Reduced leaf area (*ca.* 1.6-fold) was observed in both genotypes under −P treatment as compared to control plants (Figure 1B). Both genotypes grown in −P condition showed increased root biomass; however, BAT477 showed a 1.9-fold higher root dry weight as compared to DOR364 (*p* ≤ 0.05) (Figure 1C). The increased root biomass, as well as the arrested shoot growth observed in the P deficiency treatment, resulted in a 1.7-fold and 1.5-fold higher (*p* ≤ 0.05) root to shoot dry weight (DW) ratio in BAT477 and DOR364 plants, respectively (Figure 1D).

Similar results as those presented in Figure 1 have been reported for genotypes BAT477 and DOR364 under P deficiency during bean-rhizobia symbiotic nitrogen fixation, with respect to growth, nodulation and nitrogen fixation capacity in both field and greenhouse conditions [41,42]. Tang *et al.*[42] found that P_i_ concentration in plants under −P conditions were only 12%–30% of those grown in optimal P supply, while in our growth conditions, the concentration of P_i_ was only 16 and 13% for BAT477 and DOR364, respectively, when compared with the P_i_ concentration present in control plants. Tang *et al.*[41,42] also reported that both genotypes showed an increase in the root/shoot ratio under −P conditions (Figure 1D); however, we also found an increase in root dry weight compared with the control plants in both genotypes (Figure 1C). Our results (Figure 1) are consistent with the typical responses of different plant species to limited P, where the increase in root growth allows the plant to colonize a larger area and to increase the opportunity of acquiring soil P [7].

### 2.2. Comparative Expression Analysis of Regulatory Genes from the PvPHR1 Signaling Pathway, BAT477 vs. DOR364 P-Deficient Roots

**Figure 2:**
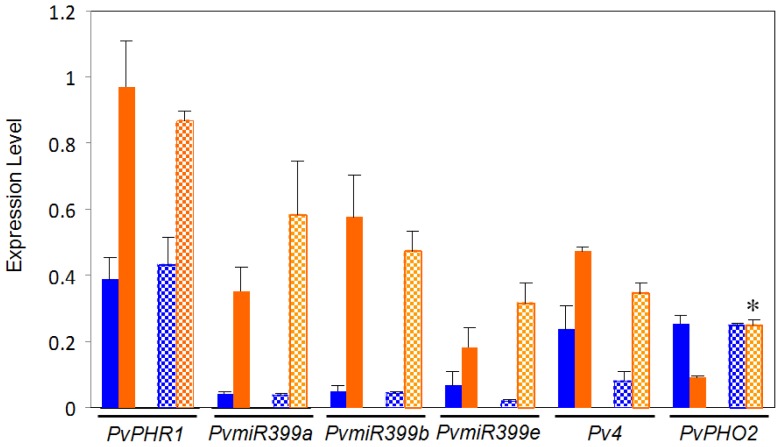
Expression of regulatory genes from the PvPHR1 signal pathway in roots of common bean BAT477 (solid bars) and DOR364 (hatched bars) genotypes. Plants were grown for three weeks under P sufficient (blue bars) or in P deficient (orange bars) conditions. Transcript levels were determined by qRT-PCR. Values are the mean (±SE) from three independent experiments with nine replicates per experiment. * Significantly different response to P deficiency between BAT477 and DOR364 (*p* ≤ 0.05).

This work aimed to investigate if possible variations in the PvPHR1 signaling pathway between the BAT477 and DOR364 common bean genotypes could be a factor related to the contrasting P deficiency response observed for these two genotypes [41]. To this end, we first performed a comparative gene expression analysis, through qRT-PCR, of the regulatory genes from this pathway that have been identified in *P. vulgaris*[36]. The regulatory genes tested were those coding for: the MYB family TF *PvRHR1*, *Pv4*, *PvPHO2 (PvUBC4)* and three isoforms of PvmiR399, hereby named PvmiR399a, PvmiR399b and PvmiR399e [48]. *PvRHR1* is a positive regulator of PvmiR399 and P-responsive genes [20,36]. *Pv*4 is a negative regulator of PvmiR399 by means of the target mimicry mechanism [33]. *PvPHO2* (*PvUBC4*) is a negative regulator of P-responsive proteins, and it is the target gene of PvmiR399 [29,36]. The analysis was performed on roots from plants grown in the P deficiency treatment and in the control condition. As expected, both genotypes showed significant increases in the transcript levels of *PvPHR1*, *Pv4* and *PvmiR399* (isoforms a, b and e) in roots from plants under −P as compared to control plants (Figure 2). The increase in *PvPHR1* expression ranged from 2.5- to 3-fold. *Pv4* increased by 4.6-fold in DOR364 and 2.0-fold in BAT477 in −P roots. A dramatic increase in the expression levels of the three isoforms of Pvmir399*,* ranging from 9.3- to 15-fold, was observed in both genotypes. Previously, we analyzed the expression of only the *PvmiR399b* isoform in P-deficient bean roots [36]; however, the other two recently detected isoforms [48] also showed a similar trend of induction under P deficiency. As expected, the transcript levels of the negative regulator *PvPHO2* decreased 3.7-fold in P-deficient roots from BAT477 plants; however, a significant difference was observed in the DOR364 genotype that showed similar transcript levels of *PvPHO2* in stress *vs.* control conditions. DOR364 −P roots showed a 2.7-fold more (*p* ≤ 0.05) *PvPHO2* transcript level than BAT477 P-deficient roots (Figure 2).

The ubiquitin E2 conjugase activity of PHO2 results in ubiquitination and subsequent degradation of target proteins, such as PHT1, a high affinity P transporter. The P deficiency response of *PHO2* transcript degradation mediated by miR399 allows the required increase in concentration of P transporters during the stress condition [3]. Transgenic plants of *Arabidopsis* expressing a *PHO2* gene where the 5′ UTR (un-translated region) was eliminated and, hence, eliminating complementary miR399 binding sites, resulted in a high and stable *PHO2* transcript levels in P-limited plants. In addition, the transgenic plants expressing the miR399-deregulated *PHO2* showed less *PHT1* transcript as compared to the wild-type under −P condition [29].

Our data show a variation in *PvPHO2* regulation of gene expression in the −P sensitive DOR364 genotype: the transcript level was as high in −P as in the control condition (Figure 2). Higher PvPHO2 content in DOR364 −P roots might lead to an increased degradation of relevant −P responsive proteins, such as the PHT1 transporter, that in turn result in a less efficient P transport and lower P content in P-deficient conditions. As shown in Figure 1, DOR364 −P roots showed less P_i_ content as compared to −P roots from BAT477 plants. We postulated that the increased expression of *PvPHO2* is a relevant factor related to DOR364 phenotype of higher sensitivity to P starvation.

### 2.3. Differential Regulation of PvPHO2 by PvmiR399 in BAT477 vs. DOR364 P-Deficient Roots

Based on the results obtained on the contrasting expression levels of *PvPHO2* between BAT477 and DOR364 in P-deficient roots, we aimed to explore factors that could explain the observed variation in the regulation of gene expression.

**Figure 3:**
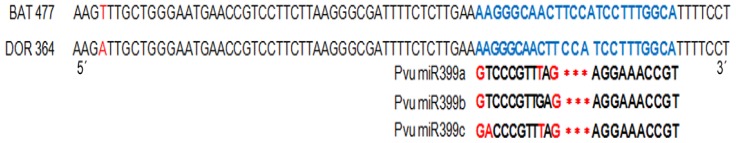
Alignment of all miR399 isoforms with the complementarity sequence of *Pv4* gene (blue) from BAT477 and DOR364 genotypes. Nucleotides displaying full base pairing are shown in black, and those displaying a mismatch are shown in red. * Corresponds to positions involved in interfering with cleavage through the mimicry mechanism (loop).

In the common bean, as well as in other plants, *PvPHO2* transcript levels are negatively regulated by PvmiR399, and the action of this miRNA is in turn modulated by *Pv4* through a target mimicry mechanism [33,36]. Our data showed that PvmiR399 and *Pv4* are induced in similar levels in −P roots from both genotypes (Figure 2); however, the effectiveness of the target mimicry regulation would not only depend in the amount of PvmiR399 and *Pv4,* but also on the level of base pair complementarity between these transcripts. To explore a possible difference related to the target mimicry mechanism, we cloned the *Pv4* gene from both genotypes and analyzed its DNA sequence. This gene is highly conserved in both genotypes, showing 99% homology. As in common bean Negro Jamapa 81 [36], the *PvmiR399* target site was identified in the *Pv4* coding region from both BAT477 and DOR394 genes, showing 100% identity between the two genotypes (Figure 3). The sequence of the *Pv4* target site for PvmiR399a, b and e has characteristics expected for the target mimicry, showing base pairing and a mismatch loop required for this mechanism [33,36]. Therefore, we can propose that the target mimicry mechanism is regulating PvmiR399 action in a similar manner in both common bean genotypes, and we cannot consider this mechanism as a significant factor contributing to their contrasting P deficiency response.

**Figure 4:**
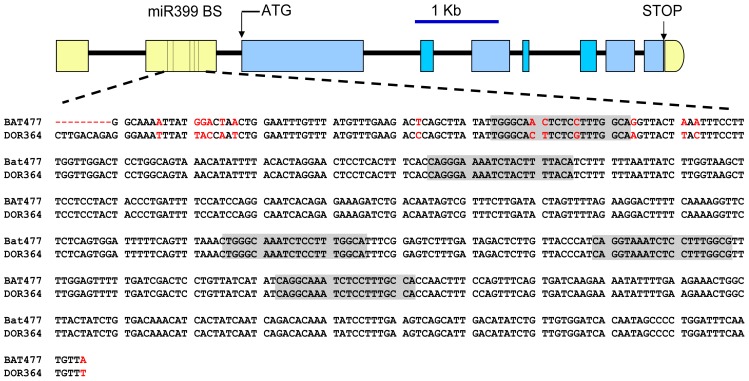
Gene structure of *PHO2* gene from the common bean. Introns are represented by black lines, and exons are represented by yellow boxes for UTRs and by blue boxes for coding regions. The ticks in the second exon of the 5′ UTR depict the position of the five predicted PvmiR399 binding sites. The 605 bp DNA sequence of the region flanking the predicted *PvmiR399* binding sites of the BAT477 and DOR364 genotypes is shown; nucleotides that differ among the two genotypes are marked in red, and the sequences complementary to PvmiR399 are shaded gray.

We then aimed to identify and analyze the sequence of the predicted target site(s) of PvmiR399 in the *PvPHO2* gene. This was done based on the *P. vulgaris* genome sequence recently made available in Phytozome (www.phytozome.net) [49,50]. As depicted in Figure 4, the *PvPHO2* gene is organized into nine exons and eight introns; two exons are contained in the 5′ UTR, and the rest in the coding region. Five predicted PvmiR399 binding sites were identified within the second exon of the *PvPHO2* 5′ UTR (Figure 4). In order to compare the DNA sequence of the PvmiR399 binding sites from the two genotypes, DNA fragments of 1.625 kb and 1.635 kb from the 5′ UTRs of BAT477 and DOR364, respectively, were cloned and sequenced. Sequence alignment of the 5′ UTR of *PvPHO2* for BAT477 and DOR364 common bean genotypes showed a number of single nucleotide polymorphisms (Figure 4). After analysis with the psRNATarget program, five 20–21 nucleotide motifs complementary to the PvmiR399 isoforms were identified, located at positions 1084, 1175, 1345, 1399, 1453 in BAT477 and 1094, 1185, 1355, 1409 and 1463 in DOR364 *PvPHO2* 5′ UTRs (Figure 4). For comparison, these predicted PvmiR399 sites will be referred to as 1 to 5, starting with the site at the 5′ end.

Our results agree with those from the *Arabidopsis PHO2* gene that contains five binding sites complementary to miR399 in its 5′ UTR region, approximately 200–400 nucleotides upstream of the start codon; miR399-dependent *PHO2* transcript cleavage at these sites has been experimentally verified [51]. Putative *PHO2* orthologs containing five miR399 binding sites in their 5′ UTRs have also been identified in rice, *Medicago truncatula* and poplar (*Populus trichocarpa*) [31]. In addition, *PHO2* gene structure is fully conserved in these diverse species, except that the fifth exon was split into two in rice [31].

In plants, the degree of base complementarity between the mRNA target site and miRNA determines the stability of miRNA:mRNA duplexes in the “RNA-induced silencing complex (RISC)”, and therefore, it is a critical characteristic for the miRNA-mediated target degradation. Based on this characteristic, a scoring system for miRNA-mRNA stability that considers mismatches, single-nucleotide bulges or gaps has been established and is widely used to predict miRNA negative regulation over the target genes. A score up to 5 is considered as functional for a stable duplex that will result in miRNA-mediated target gene degradation [25]. We analyzed the alignment score of each of the PvmiR399 isoforms with each of the *PvPHO2* putative target sites identified in the BAT477 and DOR364 genotypes (Table 1). The 30 miRNA:mRNA pairing scores obtained indicated that the five sites identified may be functional for PvmiR399 mediated *PvPHO2* mRNA degradation, except site 2 with PvmiR399b, which showed a score of 6 (Table 1). The DNA sequence and scores observed for the 2, 3, 4 and 5 PvmiR399 predicted binding sites were identical between the two genotypes (Figure 3, Table 1). However, site 1 showed three different nucleotides in the sequence (Figure 3) and clearly different PvmiR399:*PvPHO2* pairing scores when comparing BAT477 to DOR364 (Table 1).

While BAT477 showed very high complementarity of binding site 1 with the three PvmiR399 isoforms (score 1–2); the equivalent site in DOR364 genotype showed lower complementarity (score 3–4) (Table 1). The latter may indicate that PvmiR399:PvPHO2 mRNA duplexes are less stable or affect processing, thus leading to decreased miR399-mediated degradation and higher transcript level of *PvPHO2* in the DOR364 plants under P deficiency (Figure 2).

**Figure 5:**
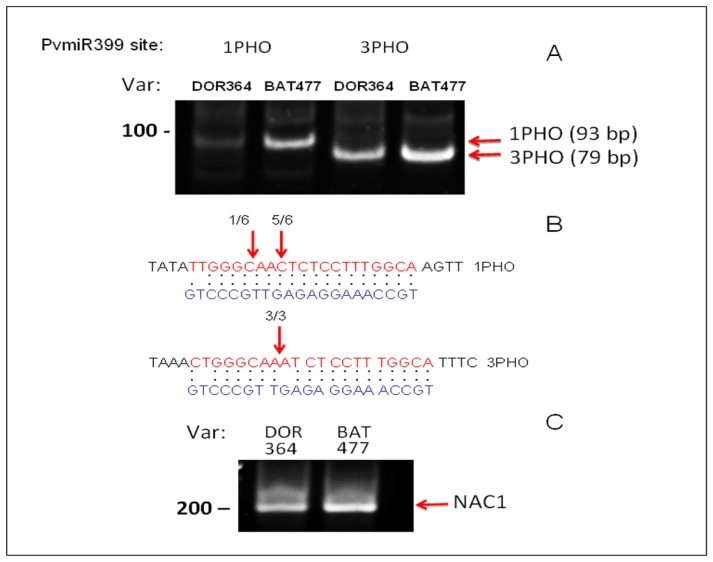
microRNA-directed cleavage of *PvPHO2* mRNA. 5′RACE analysis was performed for *PvPHO2* using oligonucleotides to specifically determine cleavage directed by PvmiR399 at two recognition sites (1 and 3) in the BAT477 and DOR364 genotypes. (**A**) PCR products corresponding to cleaved *PvPHO2* fragments from BAT477 variety were amplified by nested PCR and resolved in a 6% PA gel (arrows indicate size and position of predicted fragments; for procedure details, see the Experimental Section). The corresponding PCR fragments from BAT477 were cloned and sequenced to confirm their identity; (**B**) The alignment between *PvPHO2* binding sites 1 and 3 and PvmiR399b is shown; arrows indicate site of cleavage recovered, and numbers refer to number of independent clones analyzed. **C**. PCR fragments corresponding to cleavage of *NAC1* by miR164 that is unrelated to the P -deficiency response.

As mentioned before, four out of five PvmiR399 putative binding sites showed identical DNA sequence, while site 1 showed differences at three nucleotide positions that can potentially affect miR399 binding and/or processing. In order to explore the possibility that miR399 binding/processing is affected specifically in the DOR364 genotype, we performed modified 5′RACE experiments to assess the extent of cleavage at this site. In parallel, we also determined cleavage at the third site, which shows identical sequence between DOR364 and BAT477 genotypes, as a reference for this analysis. When compared, the abundance of a 5′RACE product at the third site, which shows identical sequence between both varieties, is similar for *PvPHO2* mRNAs in both bean genotypes (Figure 5A). In contrast, cleavage detection at PvmiR399 recognition site 1 was easily detected for BAT477, but much less so for the DOR364 genotype, which contains a less conserved sequence complementarity to miR399 (Table 1). Amplification products were purified and sequenced to confirm their identity. For binding site 1, we recovered six independent clones, where the majority (five out of six) corresponded precisely to the expected cleavage site, while for the third site, we recovered three independent clones containing the *PvPHO2* sequence cleaved exactly where predicted for miR399 recognition (Figure 5B) This result suggests that the difference in sequence at the first site corresponds to a less efficient cleavage of the *PvPHO2* mRNA directed by PvmiR399 in DOR364 genotype that can be detected by lower levels of the 5′RACE product.

## 3. Experimental Section

### 3.1. Plant Material and Growth Conditions

The common bean (*Phaseolus vulgaris*) BAT477 and DOR364 genotypes were used in this study [39]. Surface-sterilized seeds were germinated, and plants were grown in pots with vermiculite under glasshouse conditions with natural light and controlled temperature (26–28 °C). Pots were watered 3 days per week with the plant nutrient solution reported by Summerfield *et al.*[52]. For P-deficient condition (−P), cotyledons from each plant were cut 1 week after planting and K_2_HPO_4_ concentration of the plant nutrient solution was reduced from 1 mM to 5 μM. Plants were grown for 3 weeks before harvesting. Roots for nucleic acids isolation were immediately frozen in liquid nitrogen and preserved at −80 °C until used.

### 3.2. Phenotypic Characterization

The effect of P deficiency on common bean plants was assessed by measuring soluble P_i_ content in leaves, leaf area of fully expanded leaves, root dry weight and root to shoot dry weight ratio in BAT477 and DOR364 bean plants grown for 3 weeks in −P or control (full-nutrient) conditions. Soluble P_i_ content was determined using the colorimetric assay previously reported [14] on leaves that were harvested, weighed and immediately homogenized in 10 N TCA. For each parameter, 16 replicates from two independent experiments (8 replicates per experiment) were analyzed.

### 3.3. Real-Time Quantitative RT-PCR (qRT-PCR)

Total RNA was isolated from roots of bean plants grown −P or control conditions using Trizol reagent (Life Technologies, Carlsbad, CA, USA), following manufacturer instructions. For quantification of transcript levels of selected regulatory genes, cDNA was synthesized from 2 μg of total RNA using the RevertAid™ H Minus First Strand cDNA Synthesis Kit (Fermentas, Copenhagen, Denmark). For quantification of mature miRNA levels, cDNA was synthesized using the NCode miRNA First-Strand cDNA Synthesis Kit (Invitrogen, California, CA, USA). For qRT-PCR analysis, SYBR Green PCR Master Mix (Applied Biosystem, Foster City, CA, USA) and the Applied Biosystems 7500 Real-Time PCR System were used, and the thermocycler settings were: 50 °C for 2 min, 95 °C for 10 min and 40 cycles of 95 °C for 15 s and 60 °C for 60 s. The sequences of oligonucleotide primers used for each of the genes and each amplicon size are the following. For *PvPHO2*: 5′-CAGCTGCCGAAGTTTGGAA-3′ (forward) and 5′-GGGCCTGAAGAGAAAGAAGGA-3′ (reverse), 65 bp amplicon size. For *PvPHR1*: 5′-TCTGGATGCCATGGTGGTT-3′ (forward) and 5′-GCCGTTGCTTCTTGGTTGAT-3′ (reverse), 67 bp amplicon size. For *Pv4* 5′-GCTGGGAATG AACCGTCCTT-3′ (forward) and 5′-GATGGAAGTTGCCCTTTTCAAG-3′ (reverse), 59 bp amplicon size. For amplification of each miR399, 21-mer oligonucleotides were synthesized. For PvmiR399a: 5′-TGCCAAAGGAGATTTGCCCTG-3′ (forward). For PvmiR399b: 5′-TGCCAAAGGAGAGTT GCCCTG-3′ (forward). For PvmiR399e: 5′-TGCCAAAGGAGATTTGCCCAG-3′ (forward). The ubiquitin *UBC9* (TC34057) gene, which showed a constant transcript level in all the conditions tested, was included for normalization in every qRT-PCR run using the primers: 5′-GCTCTCCATTTGCTCCCTGTT-3′ (forward) and 5′-TGAGCAATTTCAGGCACCAA-3′ (reverse), amplicon size 66 bp. The qRT-PCR efficiency for each amplicon was ≥1.8. Average expression ratios (−P/C) were calculated with the ΔΔC_T_ method, as reported [14], and the fold change value (log_2_) was calculated.

### 3.4. Cloning and DNA Sequence Analysis of Pv4 Gene and of the 5′ UTR from PvPHO2

The genes encoding for *Pv4 (PvIPS1)* and for the ubiquitin E2 conjugase *PvPHO2* were identified from the common bean genome sequence deposited in Phytozome [49,50] after a BLAST search based on a EST sequence assigned to TC43701 and to TC34730, respectively (Bean Gene Index DFCI, [53]. Two primers were designed for the amplification of a 536 bp fragment from *Pv4:* 5′-CAACACTCCTTCTCAAATCCTCTC-3′ forward and 5′-AGTAAGAAGCAATTTTGTTTTG*-*3′ reverse. For the amplification of a 1.6 kb fragment from the 5′ UTR region of *PvPHO2* (locus ID Phvul.006G185400), two primers were designed: 5′-CAAACTGAAACCAAGCTTTGGGATTGA CCCTTTTC-3′ forward and 5′-AAACATTGAAATCCAGGGGTATTGTGATCC-3′ reverse. The PCR products obtained after PCR amplification from genomic DNA of the BAT477 and DOR364 genotypes were cloned in a pCR 2.1-TOPO TA vector (Invitrogen, California, CA, USA). Plasmid DNA from at least two clones from each genotype was purified and sequenced at least three times (replicates).

The 1.6 kb sequence from the *PvPHO2* 5′ UTR from each of the bean genotypes was analyzed to identify putative target sites complementary to miR399. The program psRNATarget [54] was used for such analysis. This program also allows predicting the level of matching (score) from each of the microRNA complementary sites [55].

### 3.5. Target Validation by 5′RACE of PvPHO2 mRNA

To experimentally validate targets for PvmiR399, we used a modified 5′-end rapid amplification of cDNA ends (5′RACE) approach. The 5′RACE experiment was performed, as previously described [56], using the First Choice RLM-RACE kit (Ambion, Austin, TX, USA). Total RNA (2.5 μg) was ligated to an RNA adapter using T4 RNA ligase. An aliquot was then reverse transcribed using random oligonucleotides to prime the reaction. Two rounds of nested PCR using RNA adapter-based primers and oligonucleotides designed to amplify products specific for cleavage at the first or third miR399 binding site in PvPHO2 mRNA were used: Rev1-1PHO 5′-ATGTTTACTGCCAGGAGTCC-3′ reverse; Rev2-1PHO 5′-TGAAAGTGAGGAGTTCCTAG-3′ reverse; Rev1-3PHO 5′-GGGTAAC AAGAGTCTATCAAAGACTC-3′ reverse; Rev2-3PHO 5′-CAAGAGTCTATCAAAGACTCCG-3′ reverse. Amplification products were resolved in a 6% polyacrylamide gel in 1xTBE and EtBr stained for visualization. Only after the second round of nested PCR, we obtained specific fragments after 30 or 35 cycles. For PCR product quantitation, the amplification products (after 30 cycles, in Figure 5) were analyzed using the ImageQuantTL software (GE Healthcare Bio-Sciences, Uppsala, Sweden). The PCR fragments obtained were purified by elution and ethanol precipitation from the gel and were cloned using the TOPO-TA system (Invitrogen, California, CA, USA). Several independent clones were sequenced to confirm their identity (Unidad de Secuenciación, IBT, UNAM).

## 4. Conclusions

Our analysis of two *P. vulgaris* genotypes with contrasting responses to P deficiency supports the relevant role of the *PvPHR*1/PvmiR399 signaling pathway in the response to this stress. While the transcript levels of the regulators, *PvPHR1*, *Pv4* and PvmiR399, showed similar upregulation in P-deficient roots from both genotypes, the negative regulator, *PvPHO2*, was decreased in BAT477, but not in the −P-sensitive DOR364 genotype. *PvPHO2* (E2 ubiquitin conjugase) is the target gene of PvmiR399 that induces its mRNA cleavage. From the five putative PvmiR399 binding sites identified in the *PvPHO2* 5′ UTR region of both genotypes, four sites showed identical DNA sequences, while site 1 showed three nucleotides differences between BAT477 and DOR364 genotypes. The latter was reflected in a clear difference between pairing scores among the two genotypes, thus indicating that PvmiR399:*PvPHO2* mRNA duplexes are less stable and lead to decreased PvmiR399-mediated binding and/or degradation of *PvPHO2* transcript in the DOR364 plants under P deficiency. Modified 5′RACE experiments revealed that cleavage products from *PvPHO2* site 3 (identical in both genotypes) and of *NAC1*, which is the target of miR164-independent to P deficiency, were similar in BAT477 and DOR364, while the product of *PvPHO2* site 1 was *ca.* 3-folds lower in DOR364. Our data lead us to propose that the variation in the PvmiR399-mediated regulation of *PvPHO2* between the BAT477 and DOR364 genotypes is a factor related to the contrasting response of these genotypes to P deficiency. Higher PvPHO2, resulting from less efficient PvmiR399-mediated mRNA degradation, in DOR364 would result in increased PvPHO2-mediated degradation of P-responsive proteins, such as P transporter PHT1, which would cause a decrease in P content and use efficiency in the sensitive DOR364 bean plants.

## Figures and Tables

**Table 1:** Pairing of PvmiR399 isoforms a, b and e with the predicted binding sites of the 5′ UTR of the *PvPHO2* target gene from BAT477 and DOR364 genotypes. The sequence of the predicted binding sites is shown in Figure 3. Watson-Crick base pairing is indicated by “I”; G:U base pairing by “:”; and “-” indicates a mismatch. Each miRNA:mRNA alignment score is shown in parenthesis: mismatch penalty = 1, G:U pair penalty = 0.5 [25]. Differences between BAT477 and DOR364 in binding site 1 are highlighted in red.

miRNA	*PvmiR399* binding site PHO2 5′ UTR region	BAT477 miRNA:mRNA pairing (score)	DOR364 miRNA:mRNA pairing (score)
PvmiR399a	1	I-IIIIII-IIIIIIIIIIII (2.0)	I-IIIII--IIII-IIIIIII (4.0)
PvmiR399b	1	I-IIIIIIIIIIIIIIIIIII (1.0)	I-IIIII-:IIII-IIIIIII (3.5)
PvmiR399e	1	IIIIIIII-IIIIIIIIIIII (1.0)	IIIIIII--IIII-IIIIIII (3.0)
PvmiR399a	2	IIIII-IIIIII-IIII--II (4.0)	IIIII-IIIIII-IIII--II (4.0)
PvmiR399b	2	IIIIII---III-IIII--II (6.0)	IIIIII---III-IIII--II (6.0)
PvmiR399e	2	I-III-IIIIII-IIII--II (5.0)	I-III-IIIIII-IIII--II (5.0)
PvmiR399a	3	I-IIIIIIIIIIIIIIIIIII (1.0)	I-IIIIIIIIIIIIIIIIIII (1.0)
PvmiR399b	3	I-IIIIIIIII-IIIIIIIII (2.0)	I-IIIIIIIII-IIIIIIIII (2.0)
P-miR399e	3	IIIIIIIIIIIIIIIIIIIII (0.0)	IIIIIIIIIIIIIIIIIIIII (0.0)
PvmiR399a	4	I--II:IIIIIIIIIIIIII: (3.0)	I--II:IIIIIIIIIIIIII: (3.0)
PvmiR399b	4	I--II:II-IIIIIIIIIII: (4.0)	I--II:II-IIIIIIIIIII: (4.0)
PvmiR399e	4	I--II:IIIIIIIIIIIIII: (3.0)	I--II:IIIIIIIIIIIIII: (3.0)
PvmiR399a	5	I--IIIIIIIIIIIIIII-II (3.0)	I--IIIIIIIIIIIIIII-II (3.0)
PvmiR399b	5	I--IIIII-IIIIIIIII-II (4.0)	I--IIIII-IIIIIIIII-II (4.0)
PvmiR399e	5	I--IIIIIIIIIIIIIII-II (3.0)	I--IIIIIIIIIIIIIII-II (3.0)
